# SARS-CoV-2 Omicron Variant Genomic Sequences and Their Epidemiological Correlates Regarding the End of the Pandemic: In Silico Analysis

**DOI:** 10.2196/42700

**Published:** 2023-01-10

**Authors:** Ashutosh Kumar, Adil Asghar, Himanshu N Singh, Muneeb A Faiq, Sujeet Kumar, Ravi K Narayan, Gopichand Kumar, Prakhar Dwivedi, Chetan Sahni, Rakesh K Jha, Maheswari Kulandhasamy, Pranav Prasoon, Kishore Sesham, Kamla Kant, Sada N Pandey

**Affiliations:** 1 Department of Anatomy All India Institute of Medical Sciences-Patna Patna India; 2 Etiologically Elusive Disorders Research Network New Delhi India; 3 Department of Systems Biology Columbia University Irving Medical Center New York, NY United States; 4 New York University Langone Health Center Robert I Grossman School of Medicine New York University New York, NY United States; 5 Center for Proteomics and Drug Discovery Amity Institute of Biotechnology Amity University, Maharashtra Mumbai India; 6 Dr BC Roy Multi-speciality Medical Research Centre Indian Institute of Technology Kharagpur India; 7 Department of Anatomy Institute of Medical Sciences Banaras Hindu University Varanasi India; 8 Department of Biochemistry Maulana Azad Medical College New Delhi India; 9 School of Medicine University of Pittsburgh Pittsburgh, PA United States; 10 Department of Anatomy All India Institute of Medical Sciences-Mangalagiri Mangalagiri India; 11 Department of Microbiology All India Institute of Medical Sciences-Bathinda Bathinda India; 12 Department of Zoology Banaras Hindu University Varanasi India

**Keywords:** COVID-19, pandemic, variants, immune escape, transmissibility, virulence, policy, mutations, epidemiology, data, Omicron, virus, transmission, genomic

## Abstract

**Background:**

Emergence of the new SARS-CoV-2 variant B.1.1.529 worried health policy makers worldwide due to a large number of mutations in its genomic sequence, especially in the spike protein region. The World Health Organization (WHO) designated this variant as a global variant of concern (VOC), which was named “Omicron.” Following Omicron’s emergence, a surge of new COVID-19 cases was reported globally, primarily in South Africa.

**Objective:**

The aim of this study was to understand whether Omicron had an epidemiological advantage over existing variants.

**Methods:**

We performed an in silico analysis of the complete genomic sequences of Omicron available on the Global Initiative on Sharing Avian Influenza Data (GISAID) database to analyze the functional impact of the mutations present in this variant on virus-host interactions in terms of viral transmissibility, virulence/lethality, and immune escape. In addition, we performed a correlation analysis of the relative proportion of the genomic sequences of specific SARS-CoV-2 variants (in the period from October 1 to November 29, 2021) with matched epidemiological data (new COVID-19 cases and deaths) from South Africa.

**Results:**

Compared with the current list of global VOCs/variants of interest (VOIs), as per the WHO, Omicron bears more sequence variation, specifically in the spike protein and host receptor-binding motif (RBM). Omicron showed the closest nucleotide and protein sequence homology with the Alpha variant for the complete sequence and the RBM. The mutations were found to be primarily condensed in the spike region (n=28-48) of the virus. Further mutational analysis showed enrichment for the mutations decreasing binding affinity to angiotensin-converting enzyme 2 receptor and receptor-binding domain protein expression, and for increasing the propensity of immune escape. An inverse correlation of Omicron with the Delta variant was noted (r=–0.99, *P*<.001; 95% CI –0.99 to –0.97) in the sequences reported from South Africa postemergence of the new variant, subsequently showing a decrease. There was a steep rise in new COVID-19 cases in parallel with the increase in the proportion of Omicron isolates since the report of the first case (74%-100%). By contrast, the incidence of new deaths did not increase (r=–0.04, *P*>.05; 95% CI –0.52 to 0.58).

**Conclusions:**

In silico analysis of viral genomic sequences suggests that the Omicron variant has more remarkable immune-escape ability than existing VOCs/VOIs, including Delta, but reduced virulence/lethality than other reported variants. The higher power for immune escape for Omicron was a likely reason for the resurgence in COVID-19 cases and its rapid rise as the globally dominant strain. Being more infectious but less lethal than the existing variants, Omicron could have plausibly led to widespread unnoticed new, repeated, and vaccine breakthrough infections, raising the population-level immunity barrier against the emergence of new lethal variants. The Omicron variant could have thus paved the way for the end of the pandemic.

## Introduction

### Background

A new variant of SARS-CoV-2 (lineage B.1.1.529) was reported from Botswana, South Africa, and multiple other countries [1], which the World Health Organization (WHO) designated as a global variant of concern (VOC) named “Omicron” [2]. The new variant was classified in the PANGO (Phylogenetic Assignment of Named Global Outbreak) lineage as BA.1. The presence of a large number of mutations in its genomic sequence—especially in the spike protein region, including in the host receptor-binding domain (RBD)—raised speculations that Omicron can prove to be a serious epidemiological threat and contributor to subsequent COVID-19 waves globally [3]. Multiple sublineages of Omicron were then identified with a slightly varying set of mutations [4]. These Omicron subvariants differentially affected the global population, leading to burst waves in various parts of the world [5]. Omicron is currently the predominant strain causing most of the new COVID-19 cases globally [5].

### Significance of the Study

Owing to the heterogeneity of previous infections and vaccination coverage across the global population, there has been significant ambiguity in reports on the epidemiological properties of Omicron [6-9]. Specifically, it remains unclear whether the Omicron variant has an epidemiological advantage over existing variants [8]. Many researchers have proposed that Omicron’s emergence has changed the pandemic’s evolutionary course and speculated its end [10-12]. However, contradictory views are also being presented, suggesting against any sooner end of the pandemic and the possibility of the emergence of more lethal variants as the immunity that the global population gained from previous infections and vaccines fades [13]. Therefore, we aimed to resolve the existing ambiguity over the epidemiological properties of the Omicron variant using an integrated approach combining viral genomic sequence analysis and epidemiological data. Integrating viral genomic analysis with epidemiological data is a relatively novel approach; however, its success in predicting the epidemiological properties of SARS-CoV-2 variants and the future course of the COVID-19 pandemic has been validated in recent bioinformatic studies [14,15]. The findings of this study will thus provide concrete insights into the origin and epidemiological attributes of this variant to pave the way for the end of the pandemic.

### Objectives

We performed an in silico analysis of the complete genomic sequences of the Omicron BA.1 variant available on the Global Initiative on Sharing Avian Influenza Data (GISAID) platform [16] with the primary objective of predicting the functional impact of the mutations present in this variant on virus-host interactions in terms of viral transmissibility, virulence, and immune-escape capabilities. Moreover, we assessed the relative proportion of the genomic sequences of existing SARS-CoV-2 variants, which was correlated with the rise in new COVID-19 cases in the global geographical location most affected by Omicron to understand whether the new variant had an epidemiological advantage in terms of transmissibility and virulence/lethality over existing variants.

## Methods

### Data Collection

The SARS-CoV-2 genomic sequence for the Omicron variant and other global VOCs/variants of interest (VOIs) were downloaded from the EpiCoV database of GISAID [16] using the automatic search function feeding information for geographical location, SARS-CoV-2 lineage, sample collection, and sequence reporting dates (up to December 10, 2021). The optimum length and coverage of the downloaded sequences (used for variant comparisons) were obtained by selecting the “complete sequence” and “high coverage” options in the search function.

### Data Analysis

Mutational analysis on the genomic sequences was performed, and the 3D structure of the spike protein with amino acid changes in Omicron was generated using the CoVsurver app provided by GISAID [16], employing hCoV-19/Wuhan/WIV04/2019 as the reference strain. Further, a comparative mutational analysis of Omicron with existing global VOCs/VOIs (as per the WHO) [17] was generated using the “compare lineages” function at outbreak.info [18] with GISAID as the source of genomic sequence data. The Expasy Swiss Bioinformatics portal [19] was used for protein sequence translation from the viral genomic sequences. A comparative assessment of the Omicron nucleotide and protein sequences with existing global VOCs/VOIs was performed using the National Center for Biotechnology Information (NCBI) Blast tool [20].

Furthermore, the functional impact of the mutations present at the RBD of the variants was assessed using an open analysis pipeline developed by Starr et al [21], which integrates a yeast-display platform with deep mutational scanning to determine how all possible RBD amino acid mutations affect angiotensin-converting enzyme 2 (ACE2)-binding affinity and protein expression (a correlate of protein folding stability) as compared to the wild-type SARS-CoV-2 strain [22].

The epidemiological correlates of the Omicron variant were assessed based on the comparative analysis of the genomic sequences from GISAID [16] and current epidemiological data (daily new cases and deaths) made available at Worldometer for South Africa [23], as one of the regions most strongly affected by the variant (last date of collection: December 10, 2021). The number of sequences for each SARS-CoV-2 variant was retrieved using an automatic search function feeding information for the lineage and collection dates in the EpiCoV database of GISAID for the period of October 1, 2021, to December 10, 2021. A 3-day sum of the total number of sequences was noted for each variant and their relative proportions were calculated (in percentages). Data were tabulated and the distribution of each variant was charted against the COVID-19 epidemiological data (3-day sum of new cases and deaths). Statistical analysis was performed to appreciate the changes in the relationship between the variables before and after the emergence of Omicron.

### Statistical Analysis

An expected (*E*) value ≤0 was considered significant for the sequence homology match through NCBI Blast. An E value close to 0 or below and a higher Max score indicate a higher sequence homology ranking (see [24] for further details of the statistical methods in predicting significance in similarity scores). For the mutational analysis, only the mutations present in at least 75% of sequenced samples were considered for functional characterization.

For the analysis of epidemiological data, statistical tests were performed to evaluate intergroup differences among SARS-CoV-2 variants in Microsoft Excel 2019 and the R statistical package version 4.2.2. The normality of the data was examined using the Shapiro-Wilk test. Pearson (*r*) and Spearman (*ρ*) correlation tests were performed for the normally distributed and skewed data, respectively. A correlation matrix was generated and linear regression analysis was performed between the comparing variables (presented as *r* values, ranging from 0 to 1, and 95% CIs). Results were considered statistically significant at *P*≤.05. Graphs were plotted to visualize the data trends.

### Ethical Considerations

Approval from the institutional ethics committee was precluded as publicly available/open access databases were used for this study.

## Results

### Data Summary

A total of 3604 genomic sequences of Omicron from 54 countries were uploaded on GISAID up to December 10, 2021 (see Figure S1 in Multimedia Appendix 1), which were analyzed for mutational characteristics. The mutations found were primarily condensed in the spike protein region (n=28-48) of the virus; however, frequent nonspike mutations were also noted (n=20-26). In this study, we focused on analyzing the genomic sequences of Omicron’s initially most prevalent sublineage (BA.1).

### Sequence Homology of Omicron (BA.1) With Wild-Type Strains

**Figure 1:**
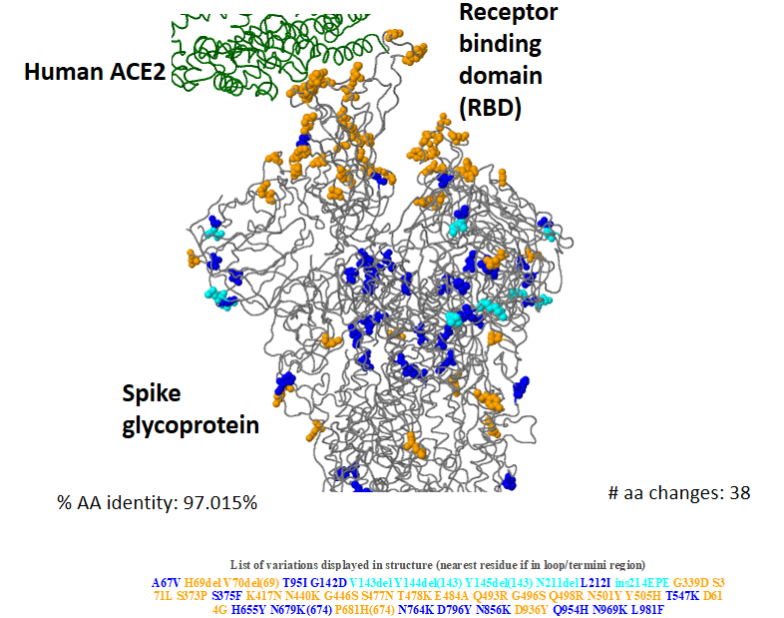
Three-dimensional structure of Omicron (BA.1) spike glycoprotein in the interaction of human angiotensin-converting enzyme 2 (ACE2), showing key amino acid substitutions. (Data source: CoVsurver app from GISAID [16]). AA/aa: amino acid.

Compared to the current list of global VOCs/VOIs (as per the WHO), Omicron showed more sequence variation, specifically in the spike protein (nucleotides 21,563-25,384; amino acids 1-1273), including the receptor-binding motif (RBM; nucleotides 22,869-23,089 and amino acids 438-508), where the riffs were most prominent (Table S1 in Multimedia Appendix 1). The homology of Omicron to the reference strain (hCoV-19/Wuhan/WIV04/2019) for the spike protein sequence varied from 96.23% to 97.8% (28-48 mutations) in the analyzed sequences (Figure 1).

### Sequence Homology of Omicron (BA.1) With Existing SARS-CoV-2 VOCs/VOIs

The analysis of Omicron’s genomic and protein sequence homology with the reference strain and current global VOCs/VOIs (as per the WHO) showed the highest similarity of Omicron with the Alpha variant for the complete sequence as well as for the RBM. However, the highest similarity for the complete nucleotide and protein sequences for the spike protein were noted with the Beta and Delta variant, respectively (see Table S1 in Multimedia Appendix 1).

### Mutational Analysis

**Figure 2:**
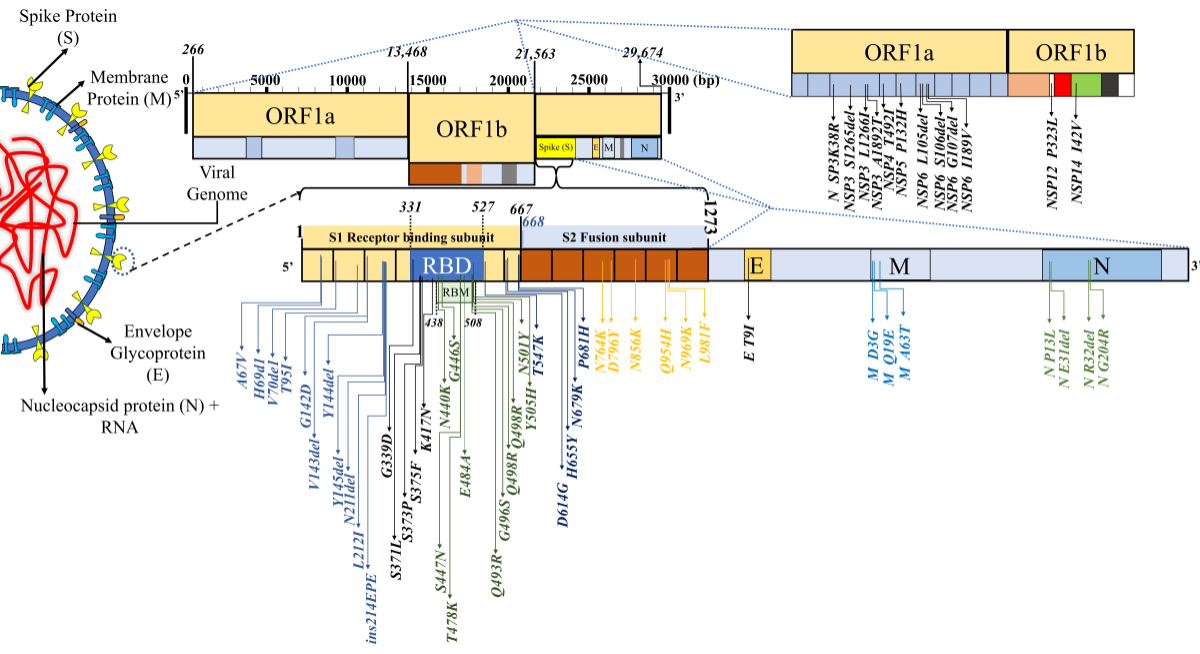
The mutational landscape in SARS-CoV-2 variant B.1.1.529 (Omicron, sublineage: BA.1). The analysis of the mutations present at the RBD using a deep mutational scanning pipeline by Starr et al [21] reflected prominent ACE2-binding affinity and protein expression changes (see Table 1). Notably, mutations decreasing the ACE2-binding affinity and protein expression were significantly greater in number. ACE2: angiotensin-converting enzyme 2; ORF: open reading frame; RBD: receptor-binding domain; RBM: receptor-binding motif.

**Figure 3:**
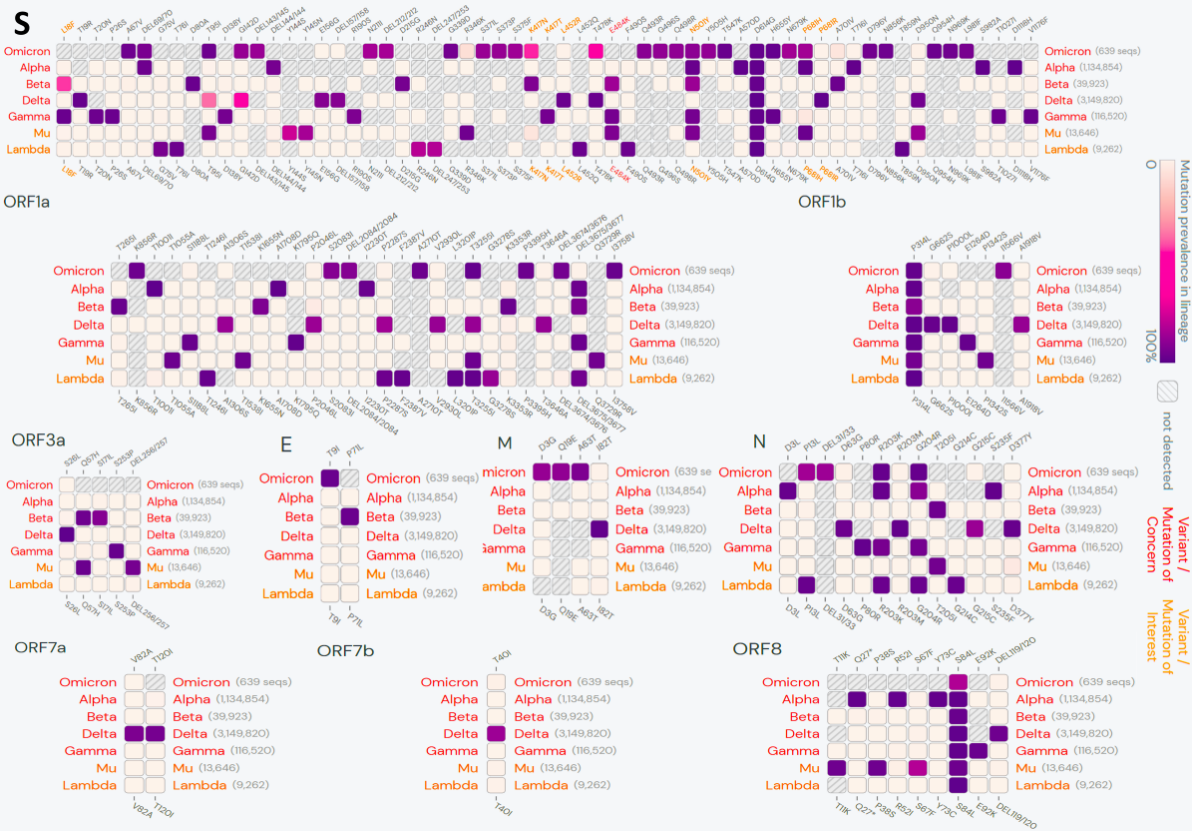
Lineage comparison between Omicron and other global variants of concerns/interest. Only mutations with >75% prevalence in at least one lineage are shown. (Data source: outbreak.info, based on the SARS-CoV-2 genomic sequences uploaded in GISAID until December 6, 2021).

Multiple clusters of closely spaced mutations were noted across the sequence, which were most densely located in the spike protein region, particularly in its S1 subunit, including the host RBM (Figure 2, Table 1). Many of the mutations in Omicron are shared with the current global VOCs/VOIs (Figure 3).

Tables 2-6 summarize the reported mutations in Omicron (BA.1) (present in at least 75% of sequences) and their functional characteristics based on the existing literature [16,21,25-41]. According to the available evidence, these mutations in PANGO lineage BA.1 can be broadly categorized into four major groups: immune escape (n=20) (Table 2), host receptor binding (n=10) (Table 3), virus replication (n=18) (Table 4), and host adaptability (n=3) (Table 5). Mutations outside of the spike protein are summarized in Table 6.

**Table 1:** Predicted impact of receptor-binding motif variations in the SARS-CoV-2 variant B.1.1.529 (Omicron, sublineage: BA.1) on interactions with the host.^a^

ACE2^b^ binding site mutations	ACE2 binding (Δlog10 KD app^c,d^)	Protein expression (Δlog mean MFI^e,f^)	ACE2 contact with SARS-CoV-2	RSA^g^ bound	SARS-CoV-1 amino acid	RaTG13 amino acid	GD Pangolin-CoV amino acid
G339D	0.06	0.30	false	0.47	G	G	G
S371L	–0.14	–0.61	false	0.46	S	S	S
S373P	–0.08	–0.22	false	0.48	F	S	S
S375F	–0.55	–1.81	false	0.48	S	S	S
K417N	–0.45	0.10	true	0.19	V	K	R
N440K	0.07	–0.12	false	0.68	N	H	N
G446S	–0.20	–0.40	true	0.55	T	G	G
S477N	0.06	0.06	false	0.76	G	S	S
T478K	0.02	0.02	false	0.48	K	K	T
E484A	–0.07	–0.23	false	0.50	P	T	E
Q493R	–0.09	–0.06	true	0.10	N	Y	Q
G496S	–0.63	0.12	true	0.04	G	G	G
Q498R	–0.06	0.10	true	0.00	Y	Y	H
N501Y	0.24	–0.14	true	0.03	T	D	N
Y505H	–0.71	0.16	true	0.12	Y	H	Y

^a^Based on the study of Starr et al [21].

^b^ACE2: angiotensin-converting enzyme 2.

^c^KD app: apparent dissociation constant.

^d^A positive Δlog10 KD app value relative to the unmutated SARS-CoV-2 receptor-binding domain (3.9 × 10^−11^ M) indicates stronger binding.

^e^MFI: mean fluorescence intensity.

^f^Positive Δlog MFI values relative to the unmutated SARS-CoV-2 receptor-binding domain indicate increased expression.

^g^RSA: relative solvent accessibility.

**Table 2:** Mutations in SARS-CoV-2 variant B.1.1.529 (Omicron, sublineage BA.1) spike protein influencing immune escape via antibody recognition sites and/or antigenic drift.^a^

Mutation	Frequency (%)^b^	Remarks	Reference
H69del	20.35	H69del+V70del have 2-fold higher infectivity compared to the wild type. H69del+V70del-containing viruses showed reduced neutralization sensitivity to mAb^c^ COVA1-21, targeting an as-yet-undefined epitope outside the RBD^d^	[16]
V70del	20.37	H69del+V70del have 2-fold higher infectivity compared to the wild type. H69del+V70del-containing viruses showed reduced neutralization sensitivity to mAb COVA1-21, targeting an as-yet-undefined epitope outside the RBD	[16]
V143del	0.12	N/A^e^	[16]
Y144del	20.94	Decreased sensitivity to convalescent sera	[25,26]
Y145del	2.33	Decreased sensitivity to convalescent sera	[16,25,26]
G339D	0.01	N/A	[16]
S371L	0.00	N/A	[16,21]
S373P	0.01	N/A	[16,21]
S375F	0.00	N/A	[16,27]
K417N	0.83	N/A	[16,21,28]
N440K	0.17	N/A	[16,21]
G446S	0.01	N/A	[16,28]
S477N	1.31	S477N was also resistant to neutralization by the human convalescent sera tested in this study, but not to vaccine-elicited sera	[16,21,29]
E484A	0.02	N/A	[27]
Q493R	0.01	N/A	[30,31]
G496S	0.01	N/A	[21]
Q498R	0.00	N/A	[16,27]
N501Y	24.11	Associated with increased transmissibility and increased affinity for human ACE2^f^ receptor	[16,21,28,32]
H655Y	2.25	N/A	[34-36]

^a^Based on the genomic sequences of Omicron uploaded on GISAID [16] (last date of collection December 10, 2021).

^b^Among all SARS-CoV-2 genomic sequences uploaded on GISAID [16].

^c^mAB: monoclonal antibody.

^d^RBD: receptor-binding domain.

^e^N/A: not applicable.

^f^ACE2: angiotensin-converting enzyme 2.

**Table 3:** Mutations in SARS-CoV-2 variant B.1.1.529 (Omicron, sublineage BA.1) spike protein influencing receptor binding.^a^

Mutation	Frequency (%)^b^	Effect on virus-host interactions	Remarks	Reference
G339D	0.01	Increased RBD^c^ expression	N/A^d^	[16,21]
S371L	0.00	Increased ACE2^e^ binding	N/A	[16,21]
S373P	0.01	Increased RBD expression	N/A	[16,21]
K417N	0.83	Increased RBD expression	N/A	[16,21,28]
N440K	0.17	Increased ACE2 binding	N/A	[16,21]
S477N	1.31	Increased ACE2 binding/ increased RBD expression	S477N was also resistant to neutralization by the human convalescent sera tested in this study, but not to vaccine-elicited sera	[16,21,29]
T478K	52.56	Increased ACE2 binding/increased RBD expression	Decreased sensitivity to convalescent sera	[21,25]
Q493R	0.01	Host change	N/A	[30,31]
G496S	0.01	Increased RBD expression	N/A	[21]
N501Y	24.11	Increased ACE2 binding/host change	Associated with increased transmissibility and increased affinity for human ACE2 receptor	[16,21,28,32]
Y505H	0.00	Increased RBD expression	N/A	[16,21]
D614G	98.51	Increased infectivity	Lower cycle threshold values were observed in G614 infections, indicating a higher viral load	[16,25,33]

^a^Based on the genomic sequences of Omicron uploaded on GISAID [16] (last date of collection December 10, 2021).

^b^Among all SARS-CoV-2 genomic sequences uploaded on GISAID [16].

^c^RBD: receptor-binding domain.

^d^N/A: not applicable.

^e^ACE2: angiotensin-converting enzyme 2.

**Table 4:** Mutations in SARS-CoV-2 variant B.1.1.529 (Omicron, sublineage BA.1) spike protein influencing viral oligomerization interfaces.^a^

Mutations	Frequency (%)^b^	Remarks	Reference
S371L	0.00	N/A^c^	[16,21]
S373P	0.01	N/A	[16,21]
S375F	0.00	N/A	[16,27]
K417N	0.83	N/A	[16,21,28]
S477N	1.31	S477N was also resistant to neutralization by the human convalescent sera tested in this study, but not to vaccine-elicited sera	[16,21,29]
Q493R	0.01	N/A	[30,31]
N501Y	24.11	Associated with increased transmissibility and increased affinity for human ACE2^d^ receptor	[16,21,28,32]
Y505H	0.00	N/A	[16,21]
N764K	0.01	N/A	[16]
D796Y	0.08	N/A	[16]
N856K	0.00	N/A	[16]
Q954H	0.00	N/A	[16,40]
N969K	0.00	N/A	[16]
L981F	0.00	N/A	[16]

^a^Based on the genomic sequences of Omicron uploaded on GISAID [16] (last date of collection December 10, 2021).

^b^Among all SARS-CoV-2 genomic sequences uploaded on GISAID [16].

^c^N/A: not applicable.

^d^ACE2: angiotensin-converting enzyme 2.

**Table 5:** Mutations in SARS-CoV-2 variant B.1.1.529 (Omicron, sublineage BA.1) spike protein influencing host adaptation and other mechanisms.^a^

Mutations	Frequency (%)^b^	Effect on virus-host interactions	Remarks	Reference
A67V	0.36	Unknown	N/A^c^	[16]
T95I	21.32	Unknown	N/A	[16]
G142D	33.40	Unknown	N/A	[16]
Q954H	0.00	Host adaptation (cell culture)	N/A	[16,40]
N211del	0.02	Unknown	N/A	[16]
L212I	0.01	Unknown	N/A	[16]
ins214EPE	0.00	Unknown	N/A	[16]
H655Y	2.25	Host adaptation (cats); spike glycoprotein fusion efficiency	N/A	[32–34]
N679K	0.09	Unknown	N/A	[16,37]
P681H	22.73	Unknown	P681H mutation at the S1/S2 site of the SARS-CoV-2 spike protein may increase its cleavability by furin-like proteases, but this does not translate into increased virus entry or membrane fusion	[16,38,39]
T547K	0.00	Unknown	N/A	[16]
N856K	0.00	Ligand binding	N/A	[16]

^a^Based on the genomic sequences of Omicron uploaded on GISAID [16] (last date of collection December 10, 2021).

^b^Among all SARS-CoV-2 genomic sequences uploaded on GISAID [16].

^c^N/A: not applicable.

**Table 6:** Mutations in SARS-CoV-2 variant B.1.1.529 (Omicron, sublineage BA.1) outside of the spike protein.^a^

Mutations		Frequency (%)^b^	Effect on virus-host interactions	Remarks	References
Envelope (E) T9I		0.09	Viral oligomerization interfaces	N/A^c^	[16]
**Membrane (M)**					
	M D3G	0.08	Unknown	N/A	[16]
	M Q19E	0.00	Unknown	N/A	[16]
	M A63T	0.01	Unknown	N/A	[16]
**Nucleocapsid (N)**					
	N P13L	0.63	Antigenic drift	P13L variant in B*27:05-restricted CD8+ nucleocapsid epitope, showing complete loss of responsiveness to the T-cell lines evaluated	[16,41]
	N E31del	0.00	Unknown	N/A	[16]
	N R32del	0.00	Unknown	N/A	[16]
	N G204R	26.20	Unknown	N/A	[16]
**Nonstructural protein (NSP)**					
	NSP3 K38R	0.01	Unknown	N/A	[16]
	NSP3 S1265del	0.02	Unknown	N/A	[16]
	NSP3 L1266I	0.02	Unknown	N/A	[16]
	NSP3 A1892T	0.00	Unknown	N/A	[16]
	NSP4 T492I	47.76	Viral oligomerization interfaces	N/A	[16]
	NSP5 P132H	0.01	Unknown	N/A	[16]
	NSP6 L105del	0.02	Unknown	N/A	[16]
	NSP6 S106del	24.74	Unknown	N/A	[16]
	NSP6 G107del	24.74	Unknown	N/A	[16]
	NSP6 I189V	0.03	Unknown	N/A	[16]
	NSP12 P323L	96.69	Viral oligomerization interfaces	N/A	[16]
	NSP14 I42V	0.00	Viral oligomerization interfaces	N/A	[16]

^a^Based on the genomic sequences of Omicron uploaded on GISAID [16] (last date of collection December 10, 2021).

^b^Among all SARS-CoV-2 genomic sequences uploaded on GISAID [16].

^c^N/A: not applicable.

### Epidemiological Correlates

**Figure 4:**
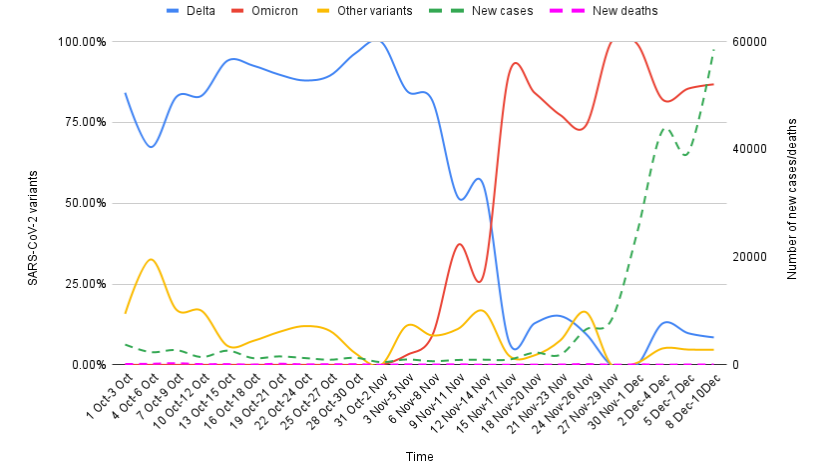
Epidemiological correlates of Omicron and Delta variants genomic sequences reported on GISAID from South Africa for the period of October 1 to December 10, 2021. The proportion of Delta and Omicron variants among the total SARS-CoV-2 genomic sequences were correlated with the new COVID-19 cases and deaths in the study period (3-day sum of each variable). A sharp change in the dominance from Delta to Omicron was observable since the report of the first Omicron case (November 5, 2021). The rise of Omicron cases paralleled the increase in the new COVID-19 cases. In comparison, the Delta variant showed a fall in the same period. Notably, there has been no increase in the number of deaths postemergence of Omicron. (Data sources: GISAID and Worldometer).

A total of 4224 SARS-CoV-2 genomic sequences (Delta, n=999; Omicron, n= 2937; and others, n= 288) were uploaded on GISAID from South Africa in the period of study. For the complete duration of the study, Delta correlated negatively with the number of new COVID-19 cases (*r*=–0.567, *P*=.004; 95% CI –0.79 to –0.21) but correlated positively with the number of new deaths (*r*=0.38, *P*=.07; 95% CI –0.025 to 0.68). The differential analysis of the SARS-CoV-2 genomic sequences from South Africa before and after the emergence of the first case of Omicron (dated November 5, 2021, EPI_ISL_7456440) reflected a sharp change in the dominance of the variant from Delta to Omicron (Figure 4). An inverse correlation of Omicron with Delta variants was noted (*r*=–0.99, *P*<.001; 95% CI –0.99 to –0.97) in the period of study. There has been a steep rise in the number of new COVID-19 cases in parallel with the increase in the proportion of Omicron since the first case of Omicron (74%-100% of total genomic sequences after November 15-17, 2021). However, no parallel increase was observed in the death cases, which otherwise showed a reverse trend (*r*=–0.04, *P*=.02; 95% CI –0.52 to 0.58) (Figure 4).

## Discussion

### Principal Findings

Our analysis of the SARS-CoV-2 genomic sequences and epidemiological data from South Africa unravels multiple observations regarding host-virus interactions, which may help to predict the further epidemiological potential of the Omicron variant. We found that compared to the current list of global VOCs/VOIs (as per the WHO), Omicron bears more sequence variation, specifically in the spike protein and RBM. Omicron showed the closest nucleotide and protein sequence homology with the Alpha variant. Further, the mutational analysis showed enrichment for the mutations decreasing ACE2-binding affinity and RBD protein expression, but increased propensity of immune escape. The analysis of the viral genomic sequences and epidemiological data from South Africa reflected an inverse correlation of Omicron with Delta variant infections, with a subsequent decrease. There was a steep rise in the number of new COVID-19 cases in parallel with the increase in the proportion of Omicron since the report of the first case; however, the incidence of deaths did not increase.

### Sequence Homology With Wild-Type Strains and Existing SARS-CoV-2 VOCs/VOIs

Our analyses showed that among the existing VOCs and VOIs, Omicron bears the highest homology of the complete sequence and RBM (nucleotide and protein sequences) with the Alpha variant (Table S1 in Multimedia Appendix 1). Interestingly, similar to Alpha variant spike gene target failure, polymerase chain reaction (PCR)-based detection is a sensitive method for detecting Omicron in clinical samples [42].

As Omicron bears key mutations from multiple existing VOCs/VOIs, with approximate sequence homology variation rather than a direct descendance, the numerous recombination events between the variants inside hosts can be a more plausible explanation for its origin.

It will be pertinent to explore the evolutionary mechanisms involved in accumulating such a large number of mutations in Omicron. Speculations were raised that the long-term persistence of SARS-CoV-2 infection in an immunocompromised host could be a probable mechanism behind the origin of Omicron [43-46]. Avanzato et al [43] and Choi et al [45] reported case studies of the persistence of infection and accumulation of novel mutations in the SARS-CoV-2 spike gene and RBD in chronically ill and immunocompromised COVID-19 patients. Another such case was reported by Karim et al [44]. The authors documented the long persistence of SARS-CoV-2 infection (for more than 6 months) in a patient with advanced HIV and antiretroviral treatment failure. Through whole-genome sequencing for SARS-CoV-2 performed at multiple time points from patient samples, the authors demonstrated the early emergence of the E484K substitution, followed by N501Y, K417T, and many other mutations (including some novel mutations) in the spike gene and RBD. An increase in the genomic diversity reflecting the intrahost evolution of SARS-CoV-2 during prolonged infection was also noted in a recent cohort study by Voloch et al [46]*.*

### Effect on Virus-Host Interactions

Our analysis shows that Omicron accumulated multiple closely spaced mutations at the RBM with ACE2 (Figure 2). Notably, this variant has many of the mutations common with the earlier VOCs (Figure 3), many of which have been shown to enhance RBD-ACE2 binding in comparison to the wild-type strain [47] (Tables 1 and 3). The selective mutations present at or near the vicinity of the RBM (N440K, S477N, T478K, and N501Y) in most of the Omicron sequences are believed to stabilize binding with ACE2 (Tables 2-3). D614G, a critical mutation in all B.1 descendants [47], is known to stabilize the trimeric structure and create a more open conformation of the RBD, allowing stronger binding with ACE2 [47]. Paradoxically, our analysis suggests that the majority of the novel or rare spike mutations (<0.2% prevalence in the total sequenced samples, Tables 2-6) in Omicron may have a deleterious effect on host interactions owing to their presence at the constrained RBD regions in terms of ACE2 binding (10/15) and/or RBD expression (8/15) (Table 3). Notably, most of the spike mutations that predicted a favorable effect on ACE2 binding, RBD expression, or both are present in current VOCs, primarily the Delta (T478K), Alpha (N501Y), and Beta (K417N) variants. Further, a set of mutations in Omicron that are present inside (P681H) or in the vicinity (D614G, H655Y) of the furin cleavage site of SARS-CoV-2 spike protein—a small stretch of peptide (PRRAR) inserted at the intersection of spike segments S1 and S2 (amino acid residues 681-685)—can enhance proteolytic cleavage of spike protein by a host protease (furin), which is considered to improve its fusion to the host cell membrane [48]. P681H is characteristically present in multiple VOCs/VOIs such as B.1.1.7, P.1, Q.1, and B.1.621 lineage variants [49]. A mutation at the exact location, P681R, has been present in the Delta variant and its emerging sublineages [50]. Characterizing the individual mutations on RBM specifies that Omicron may not have more efficient interactions with the host than existing VOCs/VOIs, specifically Delta. Further assessment of the allosteric influence and dynamic interactions of the mutations present at the RBD and other regions of spike protein and in situ/in vivo studies will be necessary to understand their exact impact on host-receptor binding and its clinical correlates. The clinical data on the severity of the disease indicated a milder illness in Omicron infection than in the existing VOCs [51,52].

### Viral Replication

Many of the mutations, especially in the nonspike regions, are linked with viral oligomerization, synthesis, and packaging of the ribonucleic acid core (Tables 4 and 6). These mutations likely have a role in virus replication inside the host cells [53]. The NSP12 P323L mutation located in the RNA-dependent RNA polymerase coding region is of particular interest (Figure 1, Table 6), as this has been a frequently observed mutation in the earlier variants (96.69%) (Table S1 in Multimedia Appendix 1). However, whether these mutations will have a positive or negative impact on viral replication remains unclear. Interestingly, the results of a comparative study [54] that employed ex vivo cultures of SARS-CoV-2 strains isolated from the respiratory tract of infected patients indicated higher replication rates for the Omicron variant. The authors observed that after 24 hours of incubation, Omicron replicated 70 times faster than wild-type and Delta variant strains in the human bronchus. In contrast, it replicated less efficiently (>10 times lower) in the human lung tissue than the wild-type strain and the replication rate was also lower than that of the Delta variant.

### Immune Escape

Most spike mutations (18/32) in Omicron have occurred at the known antibody recognition sites (Table 2). Existing studies have established the role of these mutations in immune escape against convalescent sera, vaccine-acquired antibodies, and therapeutically used monoclonal antibodies (Table 2). The evidence from in situ studies indicates potential immune escape by Omicron against convalescent sera, vaccine-acquired antibodies, and therapeutically used monoclonal antibodies [42,55,56]. Interestingly, Omicron contains the K417N and E484A mutations, which are present in multiple existing variants and are believed to contribute to immune escape [47]. Of note, the K417 locus is a known epitope for CB26, a therapeutically used monoclonal antibody in COVID-19 [47]. A more significant number of mutations in Omicron spike protein, specifically in the RBD, may be an evolutionary gain in this variant, providing it with higher immune-escape ability. Support for this notion comes from a study by Nabel et al [57], who demonstrated that SARS-CoV-2 pseudotypes containing up to seven mutations, as opposed to the one to three found in earlier VOCs, were more resistant to neutralization by therapeutic antibodies and serum from vaccine recipients [57].

A nonspike mutation in the nucleocapsid (N) protein (P13L) present in Omicron (Table 6) was shown to cause complete loss of recognition by epitope-specific (B∗27:05-restricted CD8+ nucleocapsid epitope QRNAPRITF_9-17_) T cells in a cell line–based in situ study [41]. However, no such evidence in human samples is currently available. In another study, Redd et al [58] examined peripheral blood mononuclear cell samples from PCR-confirmed, recovered/convalescent COVID-19 cases (N=30) for their anti-SARS-CoV-2 CD8+ T-cell responses with Omicron. The authors noted that only one low-prevalence (found in 7%) epitope (GVYFASTEK, restricted to HLA*A03:01 and HLA*A11:01) from the spike protein (T95I) region was mutated in Omicron [58]. The presence of these mutations raises concerns about escaping T cell immunity by Omicron [59] and hence should be explored in further detail.

The overall evidence supports Omicron’s very high immune-escape ability [42,55,56,60]. Cele et al [42] tested the ability of plasma from 14 BNT162b2-vaccinated study participants to neutralize Omicron versus the wild-type D614G virus in a live virus neutralization assay. The authors observed that Omicron showed a 41-fold decline in the 50% focus reduction neutralization test geometric mean titer compared to the wild-type D614G virus in subjects without previous infection (6/14). Interestingly, earlier, those with the infection showed relatively higher neutralization titers with Omicron (6/14), which indicated that the last infection, followed by vaccination or booster, might increase the neutralization levels and confer protection from severe disease in cases of Omicron infection.

### Epidemiological Correlates: Omicron Versus Delta Variants

The analysis of the SARS-CoV-2 genomic sequences from South Africa indicates that Omicron gained an advantage in terms of transmissibility over the Delta variant (Figure 4). A third COVID-19 wave driven by the Delta variant occurred in South Africa [61]; hence, the epidemiological characteristics of the Delta and Omicron variants in the local population should be analyzed in this backdrop. We observed that before the arrival of Omicron, the Delta variant was dominant locally; by contrast, at present, the majority of new sequences are from Omicron (Omicron vs Delta *r*=–0.99, *P*<.001; 95% CI –0.99 to –0.97) (Figure 4). The steep rise in the new COVID-19 cases in South Africa seems to be driven by Omicron, whereas Delta variant–linked cases are seeing a decline (Figure 4). The rapid rise in new COVID-19 cases connected with the emergence of a new SARS-CoV-2 variant strongly indicated the commencement of a new COVID-19 wave in South Africa [14].

Further, death, which is considered a strong indicator of virulence/lethality, showed a negative correlation (r=–0.04, *P*=.02; 95% CI –0.52 to 0.58) (Figure 4) with the rise in Omicron. However, death correlated positively with the Delta variant in the period postemergence (*r*=0.38, *P*=.07; 95% CI –0.025 to 0.68) over the complete study period. This pattern indicates that the reported incidences of death were primarily linked with Delta rather than with Omicron. The significantly reduced lethality of Omicron compared to Delta has been confirmed through recent epidemiological studies [62-64].

An approximately 2.4 (2.0-2.7) times higher transmissibility was suggested with Omicron compared to the Delta variant in the South African population [65]. An estimate from the United Kingdom indicated that Omicron’s risk of spreading the infection to members of a household is 3 times higher than that of the Delta variant [66]. A significantly shorter incubation period and early reaching of the peak have been reported for the Omicron variant [67]. Based on the epidemiological patterns observed in South Africa in our analysis, an epidemiological advantage to Omicron in comparison to Delta can be inferred in terms of transmissibility [66]. However, we found no indications of increased lethality with Omicron compared to Delta and other variants circulating in the South African population.

Notably, the presence of an immunological barrier in the population imparted by the recent COVID-19 wave mediated by the Delta variant could be a likely reason for this variant’s fall in new cases [7,68]. A continuous fall in Delta cases was also noticeable in the period before the emergence of Omicron (Figure 4), further substantiating this notion. The data records showed that a significant proportion of the local population in South Africa was fully vaccinated at the time of Omicron’s emergence (25.2%) [69]. Notably, the high number of immune escape–related mutations in Delta could have contributed to lowered efficacy of the vaccines, immunity from natural infections, and therapeutically used antibodies [47]. As Omicron contains a much higher number of immune escape–related mutations, including many shared with Delta (Figure 3), Omicron might have added potential for vaccine breakthrough infections and reinfections. Similar speculations were presented by other authors and global health regulatory bodies [2,70,71]. A higher risk of reinfections with Omicron was indicated by Pulliam et al [72] based on a retrospective analysis of routine epidemiological surveillance data to examine whether SARS-CoV-2 reinfection risk has changed over time in South Africa in the context of the emergence of the consecutive variants: Beta, Delta, and Omicron. The authors noted that as compared to the first wave driven by wild-type strains, subsequent waves by Beta and Delta variants had a lower estimated hazard ratio for reinfection versus primary infection (relative hazard ratio for wave 2 versus wave 1: 0.75, 95% CI 0.59-0.97; for wave 3 versus wave 1: 0.71, 95% CI 0.56-0.92) in comparison to Omicron (Omicron surge for the period of November 1-27, 2021, versus wave 1: 2.39, 95% CI 1.88-3.11).

### Study Limitations

We analyzed a limited number of genomic sequences and epidemiological data from specific geographical regions affected by Omicron. Further, the relative frequency of specific lineage-characterizing mutations in the Omicron variant may have varied since the study’s inception. Both of these limitations may have an impact on the quality of the results.

### Conclusion

In silico analysis of viral genomic sequences suggests that the Omicron variant has more remarkable immune-escape ability than the existing VOCs/VOIs, including Delta, but reduced virulence/lethality than other reported variants. The higher power for immune escape for Omicron was a likely reason for the resurgence in COVID-19 cases and its soon becoming a globally dominant strain. Being more infectious but less lethal than the existing variants, Omicron could have plausibly led to widespread unnoticed new, repeated, and vaccine breakthrough infections, raising the population-level immunity barrier against the emergence of new lethal variants. The Omicron variant could have thus paved the way for the end of the pandemic.

## Appendix

Multimedia Appendix 1 Global spread of the SARS-CoV-2 Omicron variant (Figure S1). Nucleotide and protein sequence homology of Omicron (BA.1) with wild-type SARS-CoV-2 and other global variants of concern/interest (Table S1).

## Abbreviations

ACE2: angiotensin-converting enzyme 2
GISAID: Global Initiative on Sharing Avian Influenza Data
NCBI: National Center for Biotechnology Information
PANGO: Phylogenetic Assignment of Named Global Outbreak
PCR: polymerase chain reaction
RBD: receptor-binding domain
RBM: receptor-binding motif
VOC: variant of concern
VOI: variant of interest
WHO: World Health Organization

## Data Sharing

Primary data used for this study are publicly available on the GISAID database [16] for SARS-CoV-2 genomic sequences and Worldometer [23] for epidemiological data. The categorized data for the study period can be availed from the corresponding author upon reasonable request.
